# A new polymorph of *N*-(2-{*N*′-[(1*E*)-2-hy­droxy­benzyl­idene]hydrazinecarbon­yl}phen­yl)benzamide

**DOI:** 10.1107/S1600536814010010

**Published:** 2014-05-10

**Authors:** Shaaban K. Mohamed, Joel T. Mague, Mehmet Akkurt, Herman Potgieter, Mustafa R. Albayati

**Affiliations:** a Chemistry and Environmental Division, Manchester Metropolitan University, Manchester M1 5GD, England; b Chemistry Department, Faculty of Science, Minia University, 61519 El-Minia, Egypt; c Department of Chemistry, Tulane University, New Orleans, LA 70118, USA; d Department of Physics, Faculty of Sciences, Erciyes University, 38039 Kayseri, Turkey; e Analytical Development Division, Manchester Metropolitan University, Manchester M1 5GD, England; f Kirkuk University, College of Science, Department of Chemistry, Kirkuk, Iraq

## Abstract

The title compound, C_21_H_17_N_3_O_3_, is a new polymorph of an already published structure [Shashidhar *et al.* (2006 ▶). *Acta Cryst.* E**62**, o4473–o4475]. The previously reported structure crystallizes in the monoclinic space group *C*2/*c*, whereas the structure reported here is in the tetra­gonal space group *I*4_1_/*a*. The bond lengths and angles are similar in both structures. The mol­ecule adopts an extended conformation *via* intra­molecular N—H⋯O and O—H⋯N hydrogen bonds; the terminal phenyl ring and the hy­droxy­lphenyl ring are twisted with respect to the central benzene ring by 44.43 (7) and 21.99 (8)°, respectively. In the crystal, mol­ecules are linked by N—H⋯O hydrogen bonds, weak C—H⋯O hydrogen bonds and weak C—H⋯π inter­actions into a three-dimensional supra­molecular network.

## Related literature   

For different medicinal functions of hydrazide–hydrazone compounds, see: Bharti *et al.* (2010 ▶); Loncle *et al.* (2004 ▶); Garoufalias *et al.* (2002 ▶); Vicini *et al.* (2002 ▶); Sondhi *et al.* (2006 ▶); Kaymakçıoğlu & Rollas (2002 ▶); Rahman *et al.* (2005 ▶); Ragavendran *et al.* (2007 ▶); Çakır *et al.* (2001 ▶); Terzioglu & Gursoy (2003 ▶); Vicini *et al.* (2009 ▶). For a monoclinic polymorph of the title compound, see: Shashidhar *et al.* (2006 ▶).
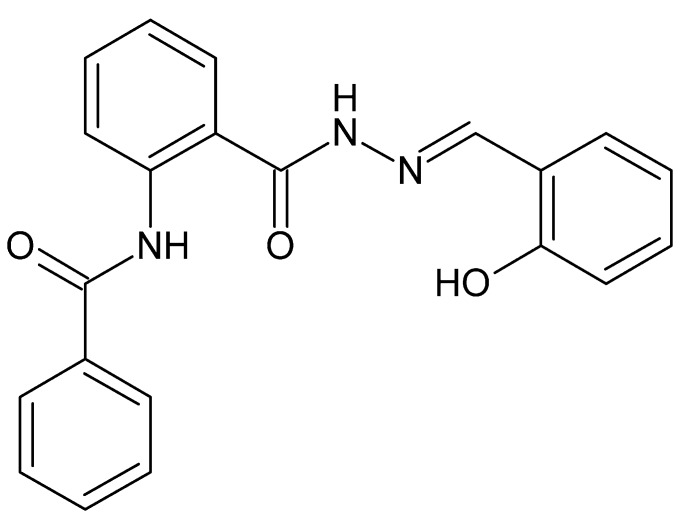


## Experimental   

### 

#### Crystal data  

C_21_H_17_N_3_O_3_
*M* *_r_* = 359.38
Tetragonal, 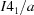

*a* = 26.7145 (14) Å
*c* = 10.1160 (5) Å
*V* = 7219.4 (8) Å^3^
*Z* = 16
Mo *K*α radiation
μ = 0.09 mm^−1^
*T* = 150 K
0.22 × 0.19 × 0.15 mm

#### Data collection  

Bruker SMART APEX CCD diffractometer
Absorption correction: multi-scan (*SADABS*; Bruker, 2013 ▶) *T*_min_ = 0.80, *T*_max_ = 0.99
37068 measured reflections
4868 independent reflections
3625 reflections with *I* > 2σ(*I*)
*R*_int_ = 0.049

#### Refinement  

*R*[*F* ^2^ > 2σ(*F* ^2^)] = 0.046
*wR*(*F* ^2^) = 0.107
*S* = 1.04
4868 reflections
244 parameters
H-atom parameters constrained
Δρ_max_ = 0.23 e Å^−3^
Δρ_min_ = −0.21 e Å^−3^

### 

Data collection: *APEX2* (Bruker, 2013 ▶); cell refinement: *SAINT* (Bruker, 2013 ▶); data reduction: *SAINT*; program(s) used to solve structure: *SHELXTL* (Sheldrick, 2008 ▶); program(s) used to refine structure: *SHELXL2014* (Sheldrick, 2008 ▶); molecular graphics: *DIAMOND* (Brandenburg & Putz, 2012 ▶); software used to prepare material for publication: *SHELXTL*.

## Supplementary Material

Crystal structure: contains datablock(s) global, I. DOI: 10.1107/S1600536814010010/xu5789sup1.cif

Structure factors: contains datablock(s) I. DOI: 10.1107/S1600536814010010/xu5789Isup2.hkl

Supporting information file. DOI: 10.1107/S1600536814010010/xu5789Isup3.cml

CCDC reference: 1000727

Additional supporting information:  crystallographic information; 3D view; checkCIF report

## Figures and Tables

**Table 1:** Hydrogen-bond geometry (Å, °) *Cg*1 is the centroid of the C1–C6 phenyl ring.

*D*—H⋯*A*	*D*—H	H⋯*A*	*D*⋯*A*	*D*—H⋯*A*
N2—H2*A*⋯O1^i^	0.91	2.02	2.9181 (15)	171
N3—H3*A*⋯O2	0.91	1.85	2.6373 (16)	143
O3—H3*B*⋯N1	0.84	1.85	2.6052 (16)	148
C4—H4⋯O2^ii^	0.95	2.60	3.3388 (19)	135
C12—H12⋯O1^i^	0.95	2.47	3.2319 (17)	137
C18—H18⋯O3^iii^	0.95	2.53	3.417 (2)	155
C17—H17⋯*Cg*1^i^	0.95	2.73	3.6561 (18)	166

Symmetry codes: (i) 
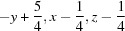
; (ii) 
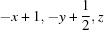
; (iii) 
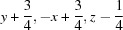
.

**Fig. 1.**
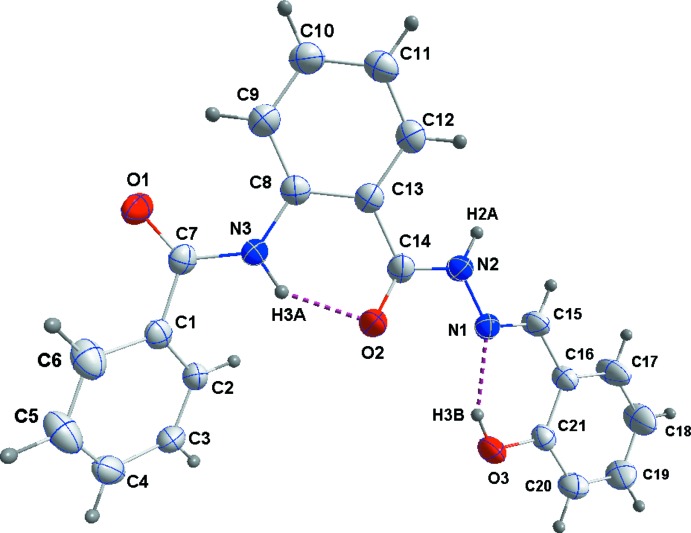
Perspective view of the title molecule with 50% probability ellipsoids. Intramolecular hydrogen bonds are shown by dotted lines.

**Fig. 2.**
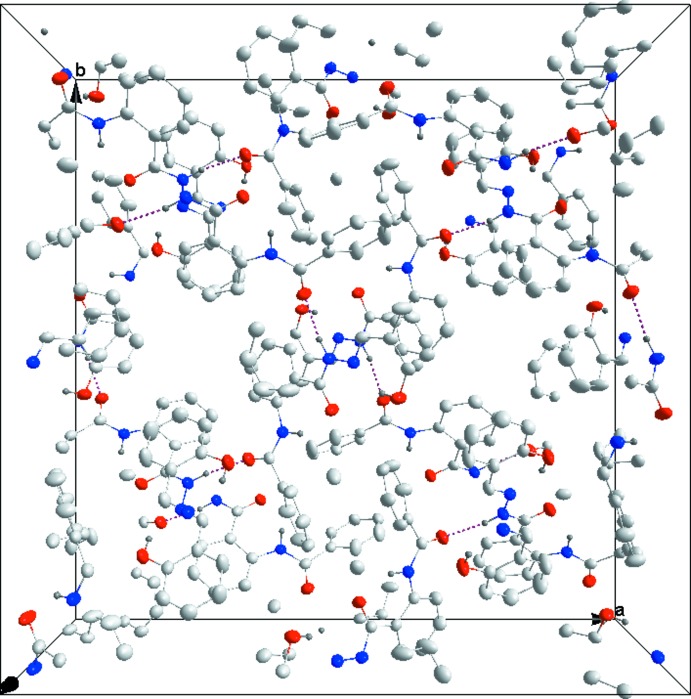
Packing viewed down the c axis with intermolecular N—H···O hydrogen bonds shown with dotted lines.

## supplementary crystallographic information

### 1. Comment

In many reports, hydrazide-hydrazone compounds are considered to be good candidates for different pharmaceutical applications such as anti-bacterial (Bharti *et al.*, 2010), antifugal (Loncle *et al.*, 2004), antimicrobial (Garoufalias *et al.*, 2002; Vicini *et al.*, 2002), anti-inflammatory (Sondhi *et al.*, 2006), anti-malarial and anti-tuberculotic activities (Kaymakçıoğlu & Rollas, 2002; Rahman *et al*., 2005) as well as anticonvulsant agents (Ragavendran *et al.*, 2007; Çakır *et al.*, 2001). In addition, hydrazide-hydrazones were reported to elicit anti-cancer (Terzioglu & Gursoy, 2003) and anti-HIV properties (Vicini *et al.*, 2009) and hence they have gained an important place in medicinal chemistry. As part of our study to obtain novel hydrazide-hydrazones with a wide spectrum of pharmaceutical properties we report herein the synthesis of the title compound as an example of a series of hydrazide-hydrazones.

The title compound, (I), is a tetragonal polymorph of the previously reported crystal structure which crystallizes in the monoclinic space group *C*2/*c* (Shashidhar *et al.*, 2006). The relative arrangement of the molecules in (I) is different from that previously reported.

The "*extended*" conformation of the title molecule (I) is largely determined by the intramolecular N—H···O and O—H···N hydrogen bonds (Table 1 and Fig. 1). The dihedral angle between the central 6-membered ring (C8–C13) and the phenyl ring (C1–C6) of the benzamide group is 44.43 (7)° while that with the phenol ring (C16–C21) is 21.99 (8)°. The molecular packing of (I) is stabilized by intermolecular N—H···O hydrogen bonds a weak C—H···π interaction (Table 1 and Fig. 2).

### 2. Experimental

In 25 ml of ethanol, a mixture of 237 mg (1 mmol) 3-amino-2-phenylquinazolin-4(3*H*)-one, 122 mg (1 mmol) of salicaldehyde and a catalytic amount of glacial acetic acid was stirred and refluxed for 6hr. The mixture was cooled and the separated solid was recrystallized from ethanol to afford the pure product as pale-yellow crystals suitable for X-ray diffraction. m.p. 497 – 501 K.

### 3. Refinement

H-atoms attached to carbon were placed in calculated positions (C—H = 0.95 Å) while those attached to nitrogen and oxygen were placed in locations derived from a difference map and their coordinates adjusted to give N—H = 0.91 and O—H = 0.84 Å. All were included as riding contributions with isotropic displacement parameters 1.2 times those of the attached atoms.

### Crystal data

**Table:**

C_21_H_17_N_3_O_3_	*D*_x_ = 1.323 Mg m^−^^3^
*M**_r_* = 359.38	Mo *K*α radiation, λ = 0.71073 Å
Tetragonal, *I*4_1_/*a*	Cell parameters from 9997 reflections
Hall symbol: -I 4ad	θ = 2.2–29.4°
*a* = 26.7145 (14) Å	µ = 0.09 mm^−^^1^
*c* = 10.1160 (5) Å	*T* = 150 K
*V* = 7219.4 (8) Å^3^	Block, pale-yellow
*Z* = 16	0.22 × 0.19 × 0.15 mm
*F*(000) = 3008	

### Data collection

**Table:**

Bruker SMART APEX CCD diffractometer	4868 independent reflections
Radiation source: fine-focus sealed tube	3625 reflections with *I* > 2σ(*I*)
Graphite monochromator	*R*_int_ = 0.049
Detector resolution: 8.3660 pixels mm^-1^	θ_max_ = 29.5°, θ_min_ = 2.2°
φ and ω scans	*h* = −36→36
Absorption correction: multi-scan (*SADABS*; Bruker, 2013)	*k* = −36→36
*T*_min_ = 0.80, *T*_max_ = 0.99	*l* = −13→13
37068 measured reflections	

### Refinement

**Table:**

Refinement on *F*^2^	0 restraints
Least-squares matrix: full	Hydrogen site location: inferred from neighbouring sites
*R*[*F*^2^ > 2σ(*F*^2^)] = 0.046	H-atom parameters constrained
*wR*(*F*^2^) = 0.107	*w* = 1/[σ^2^(*F*_o_^2^) + (0.0318*P*)^2^ + 5.367*P*] where *P* = (*F*_o_^2^ + 2*F*_c_^2^)/3
*S* = 1.04	(Δ/σ)_max_ = 0.001
4868 reflections	Δρ_max_ = 0.23 e Å^−^^3^
244 parameters	Δρ_min_ = −0.21 e Å^−^^3^

### Special details

**Table:**

Geometry. Bond distances, angles *etc*. have been calculated using the rounded fractional coordinates. All su's are estimated from the variances of the (full) variance-covariance matrix. The cell e.s.d.'s are taken into account in the estimation of distances, angles and torsion angles
Refinement. Refinement on *F*^2^ for ALL reflections except those flagged by the user for potential systematic errors. Weighted *R*-factors *wR* and all goodnesses of fit *S* are based on *F*^2^, conventional *R*-factors *R* are based on *F*, with *F* set to zero for negative *F*^2^. The observed criterion of *F*^2^ > σ(*F*^2^) is used only for calculating -*R*-factor-obs *etc*. and is not relevant to the choice of reflections for refinement. *R*-factors based on *F*^2^ are statistically about twice as large as those based on *F*, and *R*-factors based on ALL data will be even larger.

### Fractional atomic coordinates and isotropic or equivalent isotropic displacement parameters (Å^2^)

**Table:**

	*x*	*y*	*z*	*U*_iso_*/*U*_eq_	
O1	0.56564 (4)	0.41265 (4)	0.31994 (10)	0.0379 (3)	
O2	0.65352 (4)	0.27173 (4)	0.11479 (12)	0.0444 (4)	
O3	0.67750 (4)	0.17105 (4)	−0.15220 (12)	0.0461 (4)	
N1	0.72994 (4)	0.23746 (4)	−0.02418 (12)	0.0323 (3)	
N2	0.73567 (4)	0.27577 (4)	0.06534 (12)	0.0343 (4)	
N3	0.61447 (4)	0.35295 (4)	0.22122 (12)	0.0327 (3)	
C1	0.52568 (5)	0.35160 (5)	0.18534 (14)	0.0307 (4)	
C2	0.52601 (5)	0.33600 (5)	0.05509 (16)	0.0372 (4)	
C3	0.48315 (6)	0.31576 (6)	−0.00106 (17)	0.0416 (5)	
C4	0.44051 (6)	0.30914 (5)	0.07402 (17)	0.0407 (5)	
C5	0.44026 (6)	0.32438 (7)	0.20375 (18)	0.0521 (6)	
C6	0.48221 (6)	0.34632 (7)	0.25975 (16)	0.0457 (5)	
C7	0.57029 (5)	0.37577 (5)	0.24815 (14)	0.0302 (4)	
C8	0.66281 (5)	0.36385 (5)	0.26991 (14)	0.0310 (4)	
C9	0.67193 (6)	0.40316 (6)	0.35685 (16)	0.0401 (5)	
C10	0.71960 (6)	0.41150 (6)	0.40559 (16)	0.0419 (5)	
C11	0.75900 (6)	0.38104 (6)	0.36873 (15)	0.0389 (5)	
C12	0.75057 (5)	0.34228 (5)	0.28113 (14)	0.0342 (4)	
C13	0.70295 (5)	0.33311 (5)	0.22863 (14)	0.0298 (4)	
C14	0.69475 (5)	0.29129 (5)	0.13280 (15)	0.0325 (4)	
C15	0.76871 (5)	0.22376 (6)	−0.08880 (15)	0.0378 (5)	
C16	0.76600 (5)	0.18314 (5)	−0.18336 (14)	0.0333 (4)	
C17	0.80939 (6)	0.16779 (7)	−0.24892 (18)	0.0525 (6)	
C18	0.80889 (7)	0.12900 (8)	−0.33838 (18)	0.0565 (6)	
C19	0.76431 (6)	0.10478 (7)	−0.36461 (16)	0.0476 (5)	
C20	0.72072 (6)	0.11928 (6)	−0.30239 (16)	0.0416 (5)	
C21	0.72114 (5)	0.15829 (5)	−0.21138 (14)	0.0320 (4)	
H2	0.55560	0.33910	0.00390	0.0450*	
H2A	0.76580	0.29150	0.06880	0.0410*	
H3	0.48320	0.30640	−0.09170	0.0500*	
H3A	0.61360	0.32350	0.17590	0.0390*	
H3B	0.68340	0.19490	−0.10030	0.0550*	
H4	0.41160	0.29420	0.03630	0.0490*	
H5	0.41100	0.31980	0.25570	0.0620*	
H6	0.48130	0.35770	0.34870	0.0550*	
H9	0.64520	0.42450	0.38300	0.0480*	
H10	0.72530	0.43840	0.46500	0.0500*	
H11	0.79160	0.38670	0.40330	0.0470*	
H12	0.77770	0.32140	0.25590	0.0410*	
H15	0.79970	0.24040	−0.07460	0.0450*	
H17	0.84000	0.18460	−0.23130	0.0630*	
H18	0.83880	0.11900	−0.38160	0.0680*	
H19	0.76370	0.07790	−0.42610	0.0570*	
H20	0.69020	0.10250	−0.32180	0.0500*	

### Atomic displacement parameters (Å^2^)

**Table:**

	*U*^11^	*U*^22^	*U*^33^	*U*^12^	*U*^13^	*U*^23^
O1	0.0422 (6)	0.0323 (5)	0.0391 (6)	0.0078 (4)	−0.0023 (4)	−0.0068 (4)
O2	0.0287 (5)	0.0375 (6)	0.0669 (8)	−0.0050 (4)	0.0089 (5)	−0.0155 (5)
O3	0.0265 (5)	0.0485 (6)	0.0633 (8)	−0.0034 (4)	0.0024 (5)	−0.0142 (5)
N1	0.0298 (6)	0.0300 (6)	0.0370 (6)	0.0003 (4)	0.0016 (5)	−0.0009 (5)
N2	0.0283 (6)	0.0334 (6)	0.0412 (7)	−0.0039 (5)	0.0043 (5)	−0.0057 (5)
N3	0.0301 (6)	0.0276 (5)	0.0404 (7)	0.0001 (4)	0.0010 (5)	−0.0059 (5)
C1	0.0298 (6)	0.0250 (6)	0.0372 (7)	0.0026 (5)	0.0028 (6)	0.0027 (5)
C2	0.0300 (7)	0.0372 (7)	0.0444 (9)	−0.0025 (6)	0.0084 (6)	−0.0096 (6)
C3	0.0362 (8)	0.0401 (8)	0.0485 (9)	−0.0030 (6)	0.0035 (7)	−0.0153 (7)
C4	0.0318 (7)	0.0340 (7)	0.0564 (10)	−0.0049 (6)	0.0011 (7)	0.0010 (7)
C5	0.0329 (8)	0.0766 (12)	0.0467 (10)	−0.0075 (8)	0.0087 (7)	0.0117 (9)
C6	0.0364 (8)	0.0676 (11)	0.0332 (8)	−0.0010 (7)	0.0047 (6)	0.0041 (7)
C7	0.0334 (7)	0.0261 (6)	0.0310 (7)	0.0022 (5)	0.0029 (5)	0.0023 (5)
C8	0.0320 (7)	0.0292 (6)	0.0318 (7)	−0.0013 (5)	0.0013 (5)	0.0026 (5)
C9	0.0398 (8)	0.0375 (8)	0.0430 (9)	0.0015 (6)	−0.0007 (7)	−0.0071 (7)
C10	0.0448 (8)	0.0431 (8)	0.0379 (8)	−0.0041 (7)	−0.0041 (7)	−0.0067 (7)
C11	0.0355 (7)	0.0481 (9)	0.0330 (8)	−0.0050 (6)	−0.0040 (6)	0.0011 (6)
C12	0.0322 (7)	0.0374 (7)	0.0331 (7)	−0.0010 (5)	0.0033 (6)	0.0039 (6)
C13	0.0317 (7)	0.0278 (6)	0.0298 (7)	−0.0026 (5)	0.0042 (5)	0.0035 (5)
C14	0.0303 (7)	0.0275 (6)	0.0398 (8)	−0.0007 (5)	0.0047 (6)	0.0022 (6)
C15	0.0265 (7)	0.0440 (8)	0.0428 (9)	−0.0055 (6)	0.0024 (6)	−0.0054 (7)
C16	0.0270 (6)	0.0403 (8)	0.0325 (7)	−0.0001 (5)	−0.0004 (5)	−0.0015 (6)
C17	0.0299 (8)	0.0763 (12)	0.0514 (10)	−0.0046 (8)	0.0050 (7)	−0.0231 (9)
C18	0.0407 (9)	0.0768 (13)	0.0520 (11)	0.0056 (8)	0.0063 (8)	−0.0239 (9)
C19	0.0531 (10)	0.0507 (9)	0.0390 (9)	0.0032 (7)	−0.0011 (7)	−0.0118 (7)
C20	0.0407 (8)	0.0441 (8)	0.0399 (9)	−0.0047 (7)	−0.0063 (7)	−0.0033 (7)
C21	0.0284 (6)	0.0339 (7)	0.0336 (7)	0.0020 (5)	−0.0023 (5)	0.0049 (6)

### Geometric parameters (Å, º)

**Table:**

O1—C7	1.2303 (17)	C12—C13	1.4002 (19)
O2—C14	1.2326 (17)	C13—C14	1.495 (2)
O3—C21	1.3542 (17)	C15—C16	1.448 (2)
O3—H3B	0.8400	C16—C21	1.3990 (19)
N1—N2	1.3751 (16)	C16—C17	1.397 (2)
N1—C15	1.2783 (18)	C17—C18	1.376 (3)
N2—C14	1.3537 (18)	C18—C19	1.381 (3)
N3—C7	1.3560 (17)	C19—C20	1.379 (2)
N3—C8	1.4125 (17)	C20—C21	1.391 (2)
N2—H2A	0.9100	C2—H2	0.9500
N3—H3A	0.9100	C3—H3	0.9500
C1—C7	1.4970 (19)	C4—H4	0.9500
C1—C2	1.382 (2)	C5—H5	0.9500
C1—C6	1.391 (2)	C6—H6	0.9500
C2—C3	1.388 (2)	C9—H9	0.9500
C3—C4	1.381 (2)	C10—H10	0.9500
C4—C5	1.374 (2)	C11—H11	0.9500
C5—C6	1.386 (2)	C12—H12	0.9500
C8—C13	1.4137 (19)	C15—H15	0.9500
C8—C9	1.391 (2)	C17—H17	0.9500
C9—C10	1.384 (2)	C18—H18	0.9500
C10—C11	1.382 (2)	C19—H19	0.9500
C11—C12	1.381 (2)	C20—H20	0.9500
			
C21—O3—H3B	108.00	C16—C17—C18	121.69 (15)
N2—N1—C15	117.37 (11)	C17—C18—C19	119.18 (17)
N1—N2—C14	118.04 (11)	C18—C19—C20	120.59 (17)
C7—N3—C8	129.27 (12)	C19—C20—C21	120.38 (15)
C14—N2—H2A	124.00	O3—C21—C20	118.32 (13)
N1—N2—H2A	118.00	C16—C21—C20	119.78 (13)
C7—N3—H3A	118.00	O3—C21—C16	121.90 (12)
C8—N3—H3A	112.00	C1—C2—H2	120.00
C6—C1—C7	118.60 (13)	C3—C2—H2	120.00
C2—C1—C6	119.38 (13)	C2—C3—H3	120.00
C2—C1—C7	121.98 (12)	C4—C3—H3	120.00
C1—C2—C3	120.16 (14)	C3—C4—H4	120.00
C2—C3—C4	120.37 (15)	C5—C4—H4	120.00
C3—C4—C5	119.44 (15)	C4—C5—H5	120.00
C4—C5—C6	120.79 (15)	C6—C5—H5	120.00
C1—C6—C5	119.79 (15)	C1—C6—H6	120.00
N3—C7—C1	114.44 (12)	C5—C6—H6	120.00
O1—C7—N3	124.51 (13)	C8—C9—H9	120.00
O1—C7—C1	121.04 (12)	C10—C9—H9	120.00
N3—C8—C13	118.08 (12)	C9—C10—H10	120.00
N3—C8—C9	122.42 (12)	C11—C10—H10	120.00
C9—C8—C13	119.50 (13)	C10—C11—H11	120.00
C8—C9—C10	120.53 (14)	C12—C11—H11	120.00
C9—C10—C11	120.67 (15)	C11—C12—H12	119.00
C10—C11—C12	119.37 (14)	C13—C12—H12	119.00
C11—C12—C13	121.51 (13)	N1—C15—H15	120.00
C8—C13—C14	120.96 (12)	C16—C15—H15	120.00
C8—C13—C12	118.39 (12)	C16—C17—H17	119.00
C12—C13—C14	120.65 (12)	C18—C17—H17	119.00
O2—C14—N2	121.15 (13)	C17—C18—H18	120.00
O2—C14—C13	122.92 (13)	C19—C18—H18	120.00
N2—C14—C13	115.94 (11)	C18—C19—H19	120.00
N1—C15—C16	120.78 (13)	C20—C19—H19	120.00
C15—C16—C21	122.15 (12)	C19—C20—H20	120.00
C17—C16—C21	118.38 (13)	C21—C20—H20	120.00
C15—C16—C17	119.47 (13)		
			
C15—N1—N2—C14	178.84 (13)	C9—C8—C13—C12	2.4 (2)
N2—N1—C15—C16	178.99 (12)	C9—C8—C13—C14	−178.53 (13)
N1—N2—C14—O2	0.0 (2)	C8—C9—C10—C11	0.1 (2)
N1—N2—C14—C13	−179.85 (11)	C9—C10—C11—C12	0.7 (2)
C8—N3—C7—O1	2.7 (2)	C10—C11—C12—C13	0.0 (2)
C8—N3—C7—C1	−175.82 (13)	C11—C12—C13—C8	−1.6 (2)
C7—N3—C8—C9	−1.3 (2)	C11—C12—C13—C14	179.33 (13)
C7—N3—C8—C13	178.23 (13)	C8—C13—C14—O2	−22.0 (2)
C6—C1—C2—C3	0.7 (2)	C8—C13—C14—N2	157.86 (13)
C7—C1—C2—C3	−177.16 (13)	C12—C13—C14—O2	157.06 (14)
C2—C1—C6—C5	1.7 (2)	C12—C13—C14—N2	−23.07 (19)
C7—C1—C6—C5	179.57 (15)	N1—C15—C16—C17	−177.66 (15)
C2—C1—C7—O1	137.04 (15)	N1—C15—C16—C21	2.1 (2)
C2—C1—C7—N3	−44.39 (18)	C15—C16—C17—C18	179.19 (16)
C6—C1—C7—O1	−40.8 (2)	C21—C16—C17—C18	−0.5 (2)
C6—C1—C7—N3	137.75 (14)	C15—C16—C21—O3	0.5 (2)
C1—C2—C3—C4	−2.7 (2)	C15—C16—C21—C20	−179.54 (14)
C2—C3—C4—C5	2.3 (2)	C17—C16—C21—O3	−179.83 (14)
C3—C4—C5—C6	0.1 (3)	C17—C16—C21—C20	0.2 (2)
C4—C5—C6—C1	−2.1 (3)	C16—C17—C18—C19	0.4 (3)
N3—C8—C9—C10	177.83 (14)	C17—C18—C19—C20	0.2 (3)
C13—C8—C9—C10	−1.7 (2)	C18—C19—C20—C21	−0.5 (3)
N3—C8—C13—C12	−177.16 (12)	C19—C20—C21—O3	−179.64 (14)
N3—C8—C13—C14	1.9 (2)	C19—C20—C21—C16	0.4 (2)

### Hydrogen-bond geometry (Å, º)

Cg1 is the centroid of the C1–C6 phenyl ring.

**Table:**

*D*—H···*A*	*D*—H	H···*A*	*D*···*A*	*D*—H···*A*
N2—H2*A*···O1^i^	0.91	2.02	2.9181 (15)	171
N3—H3*A*···O2	0.91	1.85	2.6373 (16)	143
O3—H3*B*···N1	0.84	1.85	2.6052 (16)	148
C4—H4···O2^ii^	0.95	2.60	3.3388 (19)	135
C9—H9···O1	0.95	2.24	2.8751 (19)	123
C12—H12···O1^i^	0.95	2.47	3.2319 (17)	137
C18—H18···O3^iii^	0.95	2.53	3.417 (2)	155
C17—H17···*Cg*1^i^	0.95	2.73	3.6561 (18)	166

Symmetry codes: (i) −*y*+5/4, *x*−1/4, *z*−1/4; (ii) −*x*+1, −*y*+1/2, *z*; (iii) *y*+3/4, −*x*+3/4, *z*−1/4.
